# Supplementary material for: A protocol and novel tool for systematically reviewing the effects of mindful walking on mental and cardiovascular health
Source: PLoS One. 2021 Oct 12;16(10):e0258424. doi: 10.1371/journal.pone.0258424 (PMC8509926; doi:10.1371/journal.pone.0258424)
Supplement: S2 File — (DOCX) [file pone.0258424.s002.docx]

**S2. Comprehensive Instructions for Completing the Search Flow.**

Preparation

Members of both teams and the solo member download the reference manager RefWorks. In the search flow, each use of the action verb “record” signals the team members to enter data into the novel tool.

Step 1 (Inclusion by Title)

Team A: Team members, separately enter the search combination into Academic Search Premier, APA PsycInfo, PubMed, and SPORTDiscus. Record the total number of hits you get from each database. Decide on the eligibility of sources that your searches return based on our eligibility criteria and the sources’ titles. Save the eligible sources in RefWorks, and record the number of sources you included from each database. Remove duplicate sources obtained from more than one database, and record the total number of duplicates. Send the solo member all the eligible sources in a single RefWorks folder named “Walking - Your Name A - Inclusion by Title.”

Team B: Team members, separately enter the search combination into Google Scholar. Record the total number of hits you get. You will only have access to the first 1,000 sources (the first 100 pages, each showing 10 results). Decide on the eligibility of the 1,000 sources based on our eligibility criteria and the sources’ titles. Save the eligible sources in RefWorks, and record the number of sources you include from the database. Send the solo member all the eligible sources in a single RefWorks folder named “Walking - Your Name B - Inclusion by Title.”

Solo member: Combine both members of Team A’s “Walking - Your Name A - Inclusion by Title” RefWorks folders into a new folder called “Team A - Title Joint Folder.” Sort the sources by author to identify the unpaired sources (i.e. sources that only one member of Team A identified). Copy these sources to a new RefWorks folder called “Team A - Need Decision by Title.” Then, remove these sources from the “Team A - Title Joint Folder.” The remaining sources in “Team A - Title Joint Folder” are sources that are in agreement, meaning both members of Team A included them. Merge the paired sources and record their number. These sources will move to the next eligibility step, so rename the folder as “Team A - Inclusion by Title.” In the “Team A - Need Decision by Title” folder, independently decide on sources’ eligibility. Add the sources you deem eligible to the “Team A - Inclusion by Title” folder, progressively recording the number included.

Repeat the process above for Team B. Combine the “Team A - Inclusion by Title” folder with the “Team B - Inclusion by Title” folder to form the “Name of Solo Member - Inclusion by Title” folder. Remove any duplicates, and record their number and the total number of sources remaining.

Move the first half of the “Name of Solo Member - Inclusion by Title” folder to a folder named “First Half - Inclusion by Title.” Share this folder with Team A. Move the second half of the “Name of Solo Member - Inclusion by Title” folder to a folder named “Second Half - Inclusion by Title.” Share this folder with Team B.

Step 2 (Inclusion by Abstract)

Team A: Team members, separately access the “First Half - Inclusion by Title” folder in RefWorks. Within this folder, decide on the eligibility of sources based on our eligibility criteria and the sources’ abstracts. Record the number of sources you start with, the number you exclude, and the number you have remaining. Delete ineligible sources from the folder, and record their title, publication year, and reasons for exclusion in the sheet “Your Name - Exclusion by Abstract.” Send the solo member all the eligible sources in a single RefWorks folder named “Walking - Your Name A - Inclusion by Abstract.”

Team B: Team members, separately access the “Second Half - Inclusion by Title” folder in RefWorks. Within this folder, decide on the eligibility of sources based on our eligibility criteria and the sources’ abstracts. Record the number of sources you start with, the number you exclude, and the number you have remaining. Delete ineligible sources from the folder, and record their title, publication year, and reasons for exclusion in the sheet “Your Name - Exclusion by Abstract.” Send the solo member all the eligible sources in a single RefWorks folder named “Walking - Your Name B - Inclusion by Abstract.”

Solo member: Combine both members of Team A’s “Walking - Your Name A - Inclusion by Abstract” RefWorks folders into a new folder called “Team A - Abstract Joint Folder.” Sort the sources by author to identify the unpaired sources (i.e. sources that only one member of Team A identified). Copy these sources to a new RefWorks folder called “Team A - Need Decision by Abstract.” Then, remove these sources from the “Team A - Abstract Joint Folder.” The remaining sources In “Team A - Abstract Joint Folder” are sources that are in agreement, meaning both members of Team A included them. Merge the paired sources and record their number. These sources will move to the next eligibility step, so rename the folder as “Team A - Inclusion by Abstract.” In the “Team A - Need Decision by Abstract” folder, independently decide on sources’ eligibility. Add the sources you deem eligible to the “Team A - Inclusion by Abstract” folder, progressively recording the number included.

Repeat the process above for Team B. Combine the “Team A - Inclusion by Abstract” folder with the “Team B - Inclusion by Abstract” folder to form the “Name of Solo Member - Inclusion by Abstract” folder. Remove any duplicates, and record their number and the total number of sources remaining.

Move the first half of the “Name of Solo Member - Inclusion by Abstract” folder to a folder named “First Half - Inclusion by Abstract.” Share this folder with Team A. Move the second half of the “Name of Solo Member - Inclusion by Abstract” folder to a folder named “Second Half - Inclusion by Abstract.” Share this folder with Team B.

Step 3 (Inclusion by Full Text)

Team A: Team members, separately access the “First Half - Inclusion by Abstract” folder in RefWorks. Within this folder, decide on the eligibility of sources based on our eligibility criteria and the sources’ full texts. Record the number of sources you start with, the number you exclude, and the number you have remaining. Delete ineligible sources from the folder, and record their title, publication year, and reasons for exclusion in the sheet “Your Name - Exclusion by Full Text.” Send the solo member all the eligible sources in a single RefWorks folder named “Walking - Your Name A - Inclusion by Full Text.”

Team B: Team members, separately access the “Second Half - Inclusion by Abstract” folder in RefWorks. Within this folder, decide on the eligibility of sources based on our eligibility criteria and the sources’ full texts. Record the number of sources you start with, the number you exclude, and the number you have remaining. Delete ineligible sources from the folder, and record their title, publication year, and reasons for exclusion in the sheet “Your Name - Exclusion by Full Text.” Send the solo member all the eligible sources in a single RefWorks folder named “Walking - Your Name B - Inclusion by Full Text.”

Solo member: Combine both members of Team A’s “Walking - Your Name A - Inclusion by Full Text” RefWorks folders into a new folder called “Team A - Full Text Joint Folder.” Sort the sources by author to identify the unpaired sources (i.e. sources that only one member of Team A identified). Copy these sources to a new RefWorks folder called “Team A - Need Decision by Full Text.” Then, remove these sources from the “Team A - Full Text Joint Folder.” The remaining sources In “Team A - Full Text Joint Folder” are sources that are in agreement, meaning both members of Team A included them. Merge the paired sources and record their number. These sources will move to the next eligibility step, so rename the folder as “Team A - Inclusion by Full Text.” In the “Team A - Need Decision by Full Text” folder, independently decide on sources’ eligibility. Add the sources you deem eligible to the “Team A - Inclusion by Full Text” folder, progressively recording the number included.

Repeat the process above for Team B. Combine the “Team A - Inclusion by Full Text” folder with the “Team B - Inclusion by Full Text” folder to form the “Name of Solo Member - Inclusion by Full Text” folder. Remove any duplicates, and record their number and the total number of sources remaining.

Share the “Name of Solo Member - Inclusion by Full Text” folder with all members of Teams A and B.

Step 4 (Inclusion by Reference-Searching)

Team A: Team members, separately access the “Name of Solo Member - Inclusion by Full Text” folder in RefWorks. Use any online database to obtain the full texts of the sources. Carefully scan the sources’ references for other sources that may meet our eligibility criteria. Use any online database to obtain these other sources. Decide on the eligibility of their full texts based on our eligibility criteria. Send the solo member all the eligible sources in a single RefWorks folder named “Walking - Your Name A - Inclusion by Reference-Searching.”

Team B: Team members, separately access the “Name of Solo Member - Inclusion by Full Text” folder in RefWorks. Use any online database to obtain the full texts of the sources. Carefully scan the sources’ references for other sources that may meet our eligibility criteria. Use any online database to obtain these other sources. Decide on the eligibility of their full texts based on our eligibility criteria. Send the solo member all the eligible sources in a single RefWorks folder named “Walking - Your Name B - Inclusion by Reference-Searching.”

Solo member: Combine both members of Team A’s “Walking - Your Name A - Inclusion by Reference-Searching” RefWorks folders into a new folder called “Team A - Reference Joint Folder.” Sort the sources by author to identify the unpaired sources (i.e. sources that only one member of Team A identified). Copy these sources to a new RefWorks folder called “Team A - Need Decision by Reference-Searching.” Then, remove these sources from the “Team A - Reference Joint Folder.” The remaining sources In “Team A - Reference Joint Folder” are sources that are in agreement, meaning both members of Team A included them. Merge the paired sources and record their number. These sources will move to the next eligibility step, so rename the folder as “Team A - Inclusion by Reference-Searching.” In the “Team A - Need Decision by Reference-Searching” folder, independently decide on sources’ eligibility. Add the sources you deem eligible to the “Team A - Inclusion by Reference-Searching” folder, progressively recording the number included.

Repeat the process above for Team B. Combine the “Team A - Inclusion by Reference-Searching” folder with the “Team B - Inclusion by Reference-Searching” folder to form the “Name of Solo Member - Inclusion by Reference-Searching” folder. Remove any duplicates, and record their number and the total number of sources remaining.

Share the “Name of Solo Member - Inclusion by Reference-Searching” folder with all members of Teams A and B.

Primary Investigator/First Author: Create a new RefWorks folder by merging the “Name of Solo Member - Inclusion by Reference-Searching” folder with the “Name of Solo Member - For Inclusion by Full Text” folder. Name the new folder “Final Folder for Analysis.” This folder contains the sources that will be analyzed for the systematic review.

Data Extraction:

Team A: Enter the pertinent data from the first half of the articles included by full text into a data extraction spreadsheet. Resolve disagreements and inconsistencies in data extraction by reaching a consensus.

Team B: Enter the pertinent data from the second half of the articles included by full text into a data extraction spreadsheet. Resolve disagreements and inconsistencies in data extraction by reaching a consensus.
