# Supplementary material for: A protocol and novel tool for systematically reviewing the effects of mindful walking on mental and cardiovascular health
Source: PLoS One. 2021 Oct 12;16(10):e0258424. doi: 10.1371/journal.pone.0258424 (PMC8509926; doi:10.1371/journal.pone.0258424)
Supplement: Attachment — Submitted filename: Response to Reviewers.docx [file pone.0258424.s004.docx]

August 26, 2021

Dear Editors and Reviewers of *PLOS ONE*:

Thank you for the opportunity to revise and resubmit our manuscript. Using your feedback to revise our manuscript led to a better final product. The clarifications and comments that asked for revisions came from Reviewer #2. Below, we listed Reviewer #2’s clarifications and minor comments, followed by our responses.

Thank you for considering our revisions. I would be happy to answer any questions you have regarding our work.

Respectfully,

Dustin Davis (Corresponding Author)

Department of Kinesiology and Nutrition Sciences

School of Integrated Health Sciences

University of Nevada, Las Vegas

Las Vegas, NV 89154

Dustin.davis@unlv.edu

(314) 580-5507

Reviewer #2’s Clarifications and Comments and Our Responses

Clarifications

1. “Lines 109-113: Most meta-analyses include studies with interventions and populations that differ. The heterogeneity is something that can be evaluated statistically to determine whether or not it is appropriate to use meta-analysis (with random or fixed effects); and/or stratified meta-analyses can be conducted including more similar intervention types or population groups. I don’t know if the appropriateness of meta-analysis can/should be determined in advance at the stage of the protocol, presumably before the literature search has been conducted (so before knowing the number and characteristics of studies to be included). If, however, the inappropriateness of conducting a meta-analysis could be determined by the preliminary scoping searches mentioned in lines 95-96, it would seem to me that it would be better to state that as the reason for deciding at the protocol stage that meta-analysis will not be appropriate.”

Our Response: Thank you for this important feedback. We changed the phrasing of lines 109-114. We no longer state we will not conduct a meta-analysis. Instead, we state we did not plan a meta-analysis because the scoping searches revealed clinical heterogeneity among the relevant studies (i.e., population, intervention, comparison groups, outcomes, study settings).

2. “In Table 1, I don’t understand why the items “Frequency, intensity…”, “Location of the intervention”, and “Participant’ age, sex…” are included under Outcomes. Perhaps this information should be included only in the text, rather than in the PICOS table, or another category should be added to the PICOS table (e.g., Intervention and Participant characteristics).”

Our Response: We agree that the text you mentioned would be more appropriate in a different place in Table 1. We moved the participant characteristics we will extract to the population row and the intervention characteristics to the intervention row.

Minor Comments

1. “Lines 92-93: something is missing in this sentence (should perhaps be “we explain the novel tool that was created…”).”

Our Response: We corrected the sentence. Thank you.

2. “In the novel tool spreadsheet, the link did not work.”

Our Response: We apologize for providing you with a link that did not work. The [new link that works is here.](https://docs.google.com/spreadsheets/d/19za-lbLo416bJxgfEcduO4RO8dJMCI3_/edit?usp=sharing&ouid=101369687087328486793&rtpof=true&sd=true) The link takes you to the same file you already viewed, S3_File, that is housed on Google Drive. The other supplemental files are available in the [same Google Drive folder that houses the S3_File](https://drive.google.com/drive/folders/16-fyMkX6iIaF2XZBS4CN4SRPzNgAZPVP?usp=sharing).

3. “The reference management software, RefWorks, that the authors used, is not a free resource; so they may want to suggest alternative free/open-source reference management softwares to complement their free tool.”

Our Response: This is a good point. We updated the S2_File (the only place where RefWorks is mentioned) to suggest Mendeley and Zotero as alternative free options.
